# Identification and Characterization of a Cathepsin B Protease in the Marine Fish Parasite *Cryptocaryon irritans*

**DOI:** 10.3390/ani16213428

**Published:** 2026-10-30

**Authors:** Hongshu Chi, Ying Pan, Xiaodong Liu, Nengfeng Lin, Qiuhuan Jiang, Hui Gong, Ziping Zhang

**Affiliations:** 1 College of Animal Sciences, Fujian Agriculture and Forestry University, Fuzhou 350002, China; 2 Biotechnology Institute, Fujian Academy of Agricultural Sciences, Fuzhou 350003, China; 3 College of Marine Science, Fujian Agriculture and Forestry University, Fuzhou 350002, China

**Keywords:** *Cryptocaryon irritans*, cathepsin B, cysteine protease, cryptocaryoniasis

## Abstract

*Cryptocaryon irritans*, an obligate ectoparasite, causes cryptocaryoniasis and heavy economic losses to marine aquaculture. Cathepsin B (CatB) is highly expressed in *C. irritans* theronts and hypothesized to participate in parasite host invasion, yet its biochemical properties and pathogenic roles remain poorly defined. In this study, we performed bioinformatic analysis of the *Ci*CatB gene. Following codon optimization, *Ci*CatB was heterologously expressed in *Pichia pastoris*, and the biologically active recombinant *Ci*CatB protein was purified. Subcellular localization analysis revealed that *Ci*CatB predominantly localized in the cytoplasm of theronts, with partial signals detected at the cell membrane and in the extracellular space. Enzymatic assays showed that y*Ci*CatB hydrolyzed gelatin and type I collagen from large yellow croaker, with optimum activity at pH 5.0 and stable performance from 28 to 37 °C. Mg^2+^ increased enzyme activity, while Cu^2+^ almost completely abolished catalytic activity. Both E-64 and anti-*Ci*CatB serum inhibited enzyme activity. Functional blocking using inhibitor or antiserum markedly reduced parasite recovery following challenge. Our results suggest that *Ci*CatB may contribute to host invasion by degrading host structural proteins, advancing our understanding of *C. irritans* pathogenicity. *Ci*CatB represents a potential target for exploring control strategies against cryptocaryoniasis.

## Simple Summary

Marine fish are commonly infected by *Cryptocaryon irritans*, a protozoan parasite that causes severe, often fatal skin disease and results in substantial economic losses for mariculture globally. Effective prophylactic and therapeutic approaches for this disease remain limited. In the present study, we investigated a parasite-derived cathepsin B protease hypothesized to facilitate host tissue invasion. We heterologously expressed and purified the recombinant protein in yeast, characterized its subcellular localization within the parasite, and evaluated its biological functions via multiple biochemical and infection assays. We demonstrated that this protease can degrade structural components of fish skin, and functional blocking of its activity reduces parasite recovery after challenge. These results suggest that *Ci*CatB may contribute to parasite infection. This study advances our understanding of the pathogenic mechanisms of *C. irritans* and identifies a potential target for exploring novel control strategies for farmed marine fish, supporting sustainable aquaculture.

## 1. Introduction

*Cryptocaryon irritans* is one of the most virulent ciliate parasites, infecting almost all marine fish species and causing cryptocaryonosis (also known as white spot disease) in tropical and subtropical regions [1]. The broad host range and high virulence of *C. irritans* pose a serious threat to the sustainable development of the aquaculture industry [2,3,4,5]. Infections caused by *C. irritans* can lead to respiratory distress, osmotic imbalance, and secondary bacterial infections, ultimately resulting in high mortality [6,7]. Despite numerous studies focusing on the development of prevent and control methods, including therapeutic drugs [8], physical approaches [9], and vaccines [10,11], effective treatments to control *C. irritans* infections have yet to be developed.

*C. irritans* completes its life cycle in four developmental stages: the parasitic trophont, the off-host protomont, the reproductive tomont, and the infective theront [1,12,13]. The theront is the infective stage of the parasite, which actively seeks out and contacts new fish hosts, and burrows into the epithelium [14]. The secretion of extracellular products represents a core and prevalent strategy employed by parasitic protozoa to invade host tissues and establish successful infection. This mechanism has been well documented in *Ichthyophthirius multifiliis* and *Entamoeba histolytica*, which actively release extracellular virulence factors to break through host physical barriers and achieve colonization [15,16,17]. Theronts of *C. irritans* may secrete extracellular compounds that facilitate host invasion [18], a potential function supported by their subcellular structural features [19]. Therefore, analysis of the characteristics of extracellular compounds will provide critical insights for the development of effective control strategies against this parasite.

Previous studies have demonstrated that proteases play critical roles in the infection and development of protozoan parasites, including *Tritrichomonas foetus*, *Miamiensis avidus*, and *Ceratonova shasta* [20,21,22]. Recently, more than 100 protease transcripts were identified in the transcriptome of *C. irritans*, with certain serine and cysteine proteases being highly expressed in the theront and trophont stages [23]. The presence of proteases in *C. irritans* trophonts has been confirmed, and inhibitors targeting these proteases significantly reduced parasite infectivity both in vitro and in vivo [24]. Several proteases highly expressed in the theront and trophont stages have been shown to be essential for parasite infection, and the number of parasites recovered from fish immunized with a *C. irritans* recombinant proteins was significantly lower than that from control fish [18]. Hence, these findings indicate that proteases play pivotal roles in the infection and development of *C. irritans* and may serve as promising targets for the development of drugs and vaccines against this parasite [10,18]. However, current research focusing on proteases active during host invasion remains fragmentary, and detailed functional characterization of these proteases is therefore urgently required.

Cathepsin B belongs to the papain-like family of cysteine proteases and is characterized by its dual enzymatic activities, functioning as both an endopeptidase and an exopeptidase (carboxydipeptidase) [25]. Cathepsins have been implicated in parasite invasion. For instance, in *Schistosoma japonicum*, the cysteine protease *Sj*CB2 plays a critical role in cercarial skin invasion by degrading major proteins of the epidermis and dermis [26]. Similarly, *Trichinella spiralis* cathepsin B (*Ts*CB) is highly expressed at the larval invasive stage. Recombinant *Ts*CB can directly bind and degrade host intestinal type I collagen, a core structural component of the intestinal epithelial barrier, thus facilitating larval disruption of the host intestinal mucosal barrier during infection [27]. Moreover, cysteine proteases secreted by *T. spiralis*, including CatB, contribute to intestinal epithelial injury and play important roles during muscle larval invasion of the small intestinal mucosa [27,28,29]. Our preliminary transcriptomic data revealed that *Ci*CatB exhibits the highest transcript abundance among detected proteases in the *C. irritans* theront stage [13], suggesting that this gene may play a crucial role in host invasion. However, whether the CatB of *C. irritans* possesses similar functions remains to be elucidated.

In the present work, we performed eukaryotic expression of *Ci*CatB, which was identified from transcriptomic datasets, using the *Pichia pastoris* expression system. We further determined its subcellular localization, enzymatic biochemical properties, substrate-degrading capacity and effects on parasite infectivity. The aim of this study was to characterize the biochemical properties of *Ci*CatB and explore its potential involvement in host invasion.

## 2. Materials and Methods

### 2.1. Fish, Parasites and Animals

Healthy large yellow croaker (*Larimichthys crocea*, 15 ± 1 g) were obtained from a local farm in Ningde, Fujian Province, China. *C. irritans* was isolated from naturally infected host fish (*L. crocea*) collected from Ningde. The parasite was serially passaged and propagated on large yellow croaker, following a previously described protocol with minor modifications [2,30]. Briefly, tomonts detached from infected fish were collected from the bottom of plastic aquaria and transferred to 1 L glass beakers containing 500 mL of 0.22 μm filter-sterilized seawater. The beakers were incubated under constant aeration (1–2 L/min) at 26–28 °C to support tomont development and excystment. Most tomonts completed excystment and released infective theronts within 2–4 days post-collection. To ensure stable infectivity, newly emerged theronts were collected within 1 h after excystment for subsequent experiments. Female BALB/c mice aged 6–8 weeks were purchased from Laboratory Animal Center of Fujian University of Traditional Chinese Medicine (Fuzhou, China).

### 2.2. Bioinformatics Analysis of CiCatB

The full-length CDS sequence of the *Ci*CatB gene was obtained from NCBI Transcriptome database (Accession No. GEEV01000231.1). A series of bioinformatics analysis software and online tools, including ORF Finder (https://ncbi.leemina.moe/orffinder/, accessed on 16 October 2026), ProtParam (https://web.expasy.org/protparam/, accessed on 16 October 2026), SignalP-6.0, TMHMM-2.0, WoLF PSORT (https://wolfpsort.hgc.jp/, accessed on 16 October 2026) and InterProScan (https://www.ebi.ac.uk/interpro/search/sequence/, accessed on 16 October 2026), were used to comprehensively analyze and predict the physicochemical properties, signal peptide, transmembrane region, subcellular localization and potential functional catalytic sites of *Ci*CatB protein.

### 2.3. Expression of yCiCatB

To achieve heterologous expression in *P. pastoris* strain X33, the coding sequence for mature *Ci*CatB (excluding the native N-terminal signal-peptide sequence) was codon-optimized based on the codon usage database [31]. The optimized sequence (Figure S1), incorporating the restriction sites of the shuttle vector pPICZαB, was artificially synthesized. The fragment was then digested and ligated into the pPICZαB vector using T4 DNA ligase. The resulting recombinant plasmid, pPICZαB-*Ci*CatB, was linearized with Sac I and transformed into competent *P. pastoris* X33 cells by electroporation following the manufacturer’s protocol (Invitrogen, Waltham, MA, USA). Positive transformants were selected on YPD plates containing 100 μg/mL Bleomycin and subsequently verified by PCR.

Positive *P. pastoris* X33 was initially cultured in BMGY medium at 30 °C for 16–18 h until the OD600 reached 2–3. Cells were then harvested and transferred to BMMY medium for subsequent induction. Protein expression was induced by the addition of 0.5% (*v*/*v*) methanol every 12 h for 72 h. The result of induction was tested by SDS-PAGE, and Western blotting to confirm recombinant protein expression. Following purification by His-Bind resin chromatography (Novagen, Madison, WI, USA), the target protein was subjected to buffer exchange against PBS (pH 7.2) via dialysis and concentrated using a centrifugal concentrator. Total protein concentration was determined using the BCA Protein Assay Kit (Thermo Scientific, Rockford, IL, USA), and the purified protein were stored at −80 °C for subsequent analyses.

### 2.4. Gelatin Zymography Assay

Gelatin zymography assay was performed according to previously reported protocols [24,28], with slight modifications. After mixing with sample buffer, the purified protein was loaded onto a 10% polyacrylamide gel containing 0.1% gelatin (*w*/*v*) and separated by electrophoresis. Following electrophoresis, the gel was washed in 2.5% (*v*/*v*) Triton X-100 (renaturing buffer) with gentle shaking on a rocking shaker for 60 min at room temperature to allow renaturation of SDS-denatured proteins. The gel was then immersed in incubation buffer (0.1 M sodium acetate buffer pH 5.5, 1 mM EDTA, 5 mM dithiothreitol) and incubated at 37 °C with gentle shaking for 36 h. Following incubation, the gel was stained with Coomassie Brilliant Blue solution (Sangon Biotech Co., Shanghai, China) under constant agitation for 2 h. Finally, the gel was destained in a solution containing 7% acetic acid and 5% methanol for 1 h to visualize gelatinolytic activity as clear bands against a blue background.

### 2.5. Preparation of Mouse Anti-yCiCatB Polyclonal Serum

Purified y*Ci*CatB protein was used as the immunogen for the preparation of polyclonal antiserum. Ten healthy BALB/c mice were used for antiserum production. Prior to immunization, these mice were pre-bled to collect non-immune mouse serum, which was used exclusively as the negative control for immunofluorescence assays. For primary immunization, each mouse was subcutaneously injected with 50 μg purified y*Ci*CatB protein emulsified with an equal volume of complete Freund’s adjuvant. Subsequent booster immunizations were performed at 2-week intervals, where each mouse received 30 μg recombinant *Ci*CatB emulsified with incomplete Freund’s adjuvant. Serum antibody titers were detected by indirect ELISA 7 days after each booster immunization. When the antibody titer reached the expected level, whole blood was collected from the mice. The blood samples were incubated at 37 °C for 1 h and then centrifuged at 3000 rpm for 10 min at 4 °C to separate the supernatant. The obtained anti-y*Ci*CatB immune serum was aliquoted and stored at −20 °C for subsequent experimental analysis.

### 2.6. Localization of Natural CiCatB in Theronts of C. irritans

Indirect immunofluorescence analysis was carried out in *C. irritans* theronts to determine the localization of the *Ci*CatB protein. The theronts were fixed with 4% paraformaldehyde at room temperature for 30 min, washed three times with PBS, and then immobilized on poly-L-lysine-coated slides at 4 °C for 30 min. Sample slides were incubated with primary antibody (anti-y*Ci*CatB serum, diluted 1:2000) at 37 °C for 1 h. Negative control samples were treated with non-immune mouse serum at the same dilution ratio instead of the primary antibody to exclude non-specific binding. After three PBS washes, all slides were incubated with FITC-conjugated anti-mouse IgG (1:100; Sigma, St. Louis, MO, USA) at 37 °C for 1 h. Nuclei were counterstained with DAPI. Finally, the slides were mounted and observed using a Leica TCS SP8 laser scanning confocal microscope system (Leica, Wetzlar, Germany).

### 2.7. Enzyme Activity Assays

The enzyme activity of y*Ci*CatB was measured fluorometrically using the fluorogenic substrate Z-Arg-Arg-AMC (Sigma-Aldrich, St. Louis, MO, USA) to the previous report [32,33]. Refolded y*Ci*CatB was pre-activated in activation buffer (100 mM sodium acetate buffer, pH 5.0, containing 2 mM DTT and 1 mM EDTA) at 37 °C for 30 min. The activated protein was then diluted to a final concentration of 50 μg/mL with sodium acetate buffer (100 mM, pH 5.5).

The effects of pH, temperature, and metal ions on the enzymatic activity of *Ci*CatB were evaluated. To determine the optimal pH, the enzyme and substrate were incubated in buffers at various pH values (3, 4, 5, 5.5, 6, 6.5, 7, and 8). To assess the influence of temperature, reactions were carried out at pH 5.5 under different temperatures (16, 28, 32, 37, 45, 55, and 65 °C) for 1 h in the dark. To examine the effect of metal ions, individual metal ions (Ca^2+^, Ni^2+^, Cu^2+^, Fe^2+^, Mg^2+^, and Mn^2+^) were added to the activated enzyme reaction mixture at a final concentration of 0.5 mM and incubated at 37 °C for 1 h. Fluorescence intensity was measured using a microplate reader at excitation and emission wavelengths of 355 nm and 460 nm, respectively. The control group without additional ions was defined as 100% enzymatic activity. Each assay was performed in triplicate.

To confirm the specificity of the observed enzyme activity, inhibition assays were performed using the cysteine protease inhibitor E-64 and anti-*Ci*CatB immune serum. For chemical inhibition, activated y*Ci*CatB (50 μg/mL) was pre-incubated with or without E-64 (10 μM) at 37 °C for 30 min, followed by the addition of the substrate Z-Arg-Arg-AMC. After further incubation at 37 °C for 1 h, the enzyme activity was determined by measuring fluorescence intensity as described above. For antibody inhibition, activated y*Ci*CatB (10 μg) was incubated with anti-y*Ci*CatB immune serum at various dilutions (1:1000, 1:500, 1:200) at 37 °C for 1 h. The enzyme activity was then measured by fluorescence detection following a 1 h reaction with Z-Arg-Arg-AMC. Enzyme activity in the absence of immune serum was defined as 100%. Each assay was performed in triplicate.

An AMC standard curve was generated to quantify substrate hydrolysis. Briefly, 300 μL of serially diluted AMC solutions (100–1000 nM) were prepared in pH 5.5 acetic acid-sodium acetate buffer under dark conditions. Each concentration was analyzed in triplicate black wells. Fluorescence intensity (RFU) was detected at 355 nm excitation and 460 nm emission, and the averaged readings were used to fit the standard curve using GraphPad Prism.

For kinetic analysis, activated recombinant *Ci*CatB (50 μg/mL) was reacted with gradient concentrations of Z-Arg-Arg-AMC (25–200 μM) in pH 5.5 buffer. Fluorescence signals (excitation 355 nm, emission 460 nm) were continuously recorded at multiple time-points (0, 10, 20, 30, 40, 60 min). The initial reaction velocity for each substrate concentration was calculated from the slope of the linear segment of product-generation versus time curve. Product formation rates were converted based on an AMC standard curve. Km and Vmax were determined by non-linear regression fitting to the Michaelis-Menten model using GraphPad Prism software. The Lineweaver-Burk double-reciprocal plot was generated as an inset for illustrative visualization only.

### 2.8. Degradation of Host Type I Collagen Protein In Vitro

To evaluate the potential of *Ci*CatB to degrade host proteins in vitro, we tested its activity against collagen isolated from the skin of large yellow croaker. The Collagen was extracted using an ultrasonic-assisted acetic acid method [34]. Briefly, pretreated skin pieces (5 mm × 5 mm) were soaked in 0.5 M acetic acid and subjected to ultrasonication at 300 W for 11 min with a probe frequency of 20 kHz while maintaining the temperature below 20 °C. Following ultrasonication, the mixture was stirred at 20 °C for 24 h and then centrifuged at 10,000× *g* for 20 min at 4 °C. The resulting supernatant was subjected to salting-out by adding NaCl to a final concentration of 2.5 mol/L. After standing at 4 °C for 24 h, the precipitate was collected by centrifugation, dissolved in 0.5 M acetic acid, and clarified again by centrifugation. The obtained solution was dialyzed sequentially against 0.1 M acetic acid and deionized water. Finally, the dialyzed solution was concentrated by ultrafiltration using a centrifugal filter unit at 6000× *g* for 45 min at room temperature, and the retained collagen solution was collected for further use.

To assess the proteolytic activity of *Ci*CatB against host protein, the extracted collagen was incubated with activated enzyme (at a 1:6 ratio) in pH 5.5 buffer at 37 °C for 1 and 2 h. Several control groups were included: collagen alone (without recombinant protein), heat-inactivated *Ci*CatB, and enzyme pre-treated with the cysteine protease inhibitor E-64. Heat inactivation was performed by incubating 50 μL of activated *Ci*CatB at 95 °C for 5 min. For inhibitor treatment, activated *Ci*CatB was pre-incubated with 10 μM E-64 at 37 °C for 30 min prior to substrate addition. After incubation, samples were mixed with loading buffer, boiled for 5 min, and analyzed by SDS-PAGE.

### 2.9. Effects of Protease Inhibitor E-64 and Anti-CiCatB Serum on C. irritans Theront Infectivity

Large yellow croakers (mean body weight = 15 ± 1 g) were used as experimental animals to evaluate the protective effects of antiserum and inhibitors against *C. irritans* infection. Prior to the experiment, the experimental fish were confirmed to be negative for *C. irritans* infection. Theronts were allocated into three groups and incubated with aeration (5 L/min) for 1 h under different treatments: mouse anti-y*Ci*CatB serum (1:1000 dilution, antiserum group), 10 μM E-64 (inhibitor group), and an equal volume of PBS (control group). After treatment, theronts from each group were used to challenge large yellow croaker by immersion at a dose of 2500 theronts per fish. Each challenge group was performed in triplicate, with five fish per replicate. Following challenge, each fish was transferred into its own separate 15 L clean tank. All tanks were continuously aerated with moderate light shielding to facilitate complete detachment of tomonts from fish bodies. Since each fish was housed individually, tomonts were collected and enumerated for each individual fish. At day 5 post-challenge, the parasite recovery rate was calculated as the percentage of tomonts recovered from each fish relative to the initial infective dose of 2500 theronts.

### 2.10. Statistical Analysis

Data were analyzed and graphed using GraphPad Prism 8.0.2. Data were presented as mean ± standard deviation (SD). For in vitro protease relative-activity, one-way ANOVA with Dunnett’ s test was used for comparisons of each ion-treatment versus blank control if data met normality and equal-variance criteria; otherwise, the Kruskal–Wallis test coupled with Dunn’ s test was performed. For in vivo parasite recovery rate data with skewed distribution, the Kruskal–Wallis test followed by Dunn’ s test was used to compare each treatment group against the control [35]. Statistical significance was defined as *p* < 0.05.

## 3. Results

### 3.1. Bioinformatics Analysis of CiCatB

The CDS of *Ci*CatB is 1359 bp in length, encoding a predicted protein of 452 amino acids with a molecular mass of approximately 50.9 kDa and a theoretical isoelectric point (pI) of 5.59. *Ci*CatB does not contain a transmembrane region, but it has a signal peptide. Subcellular localization prediction suggests a secretory pathway, indicating that *Ci*CatB is likely a secretory protein. Homology alignment and evolutionary conservation analysis indicated that *Ci*CatB belonged to the C1A cysteine protease family and contained the canonical GCNGG motif. The putative catalytic triad of *Ci*CatB was identified as Cys245–His390–Asn410, and the conserved oxyanion hole residue was Gln239. *Ci*CatB possessed the typical sequence characteristics of functional cathepsin B.

### 3.2. Expression and Zymography of yCiCatB

**Figure 1:**
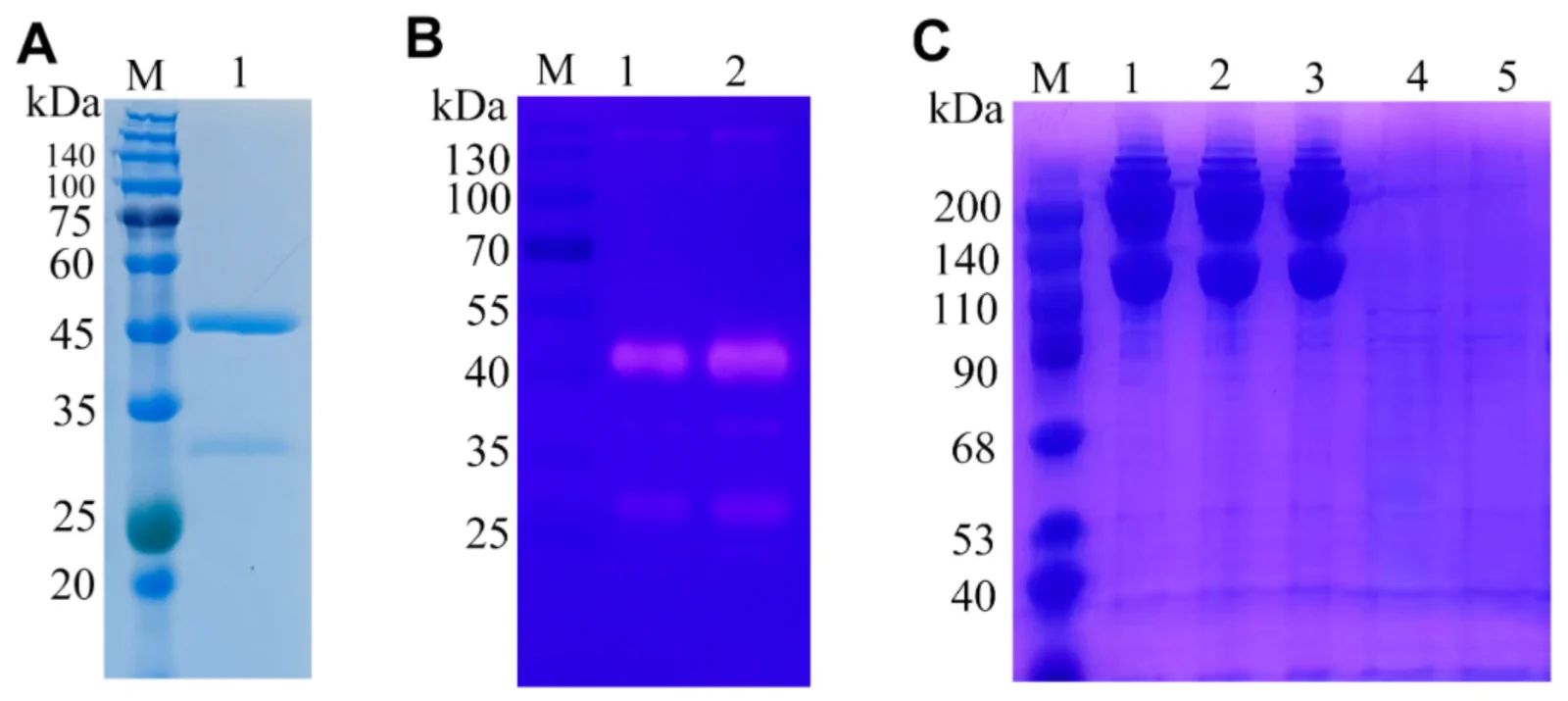
Expression and zymography analyses of y*Ci*CatB. (**A**) SDS-PAGE gel showing y*Ci*CatB. Lane M, protein marker; lane 1, purified y*Ci*CatB. (**B**) Gelatin zymography was performed followed by Coomassie brilliant blue staining. A clear degradation band at approximately 50 kDa indicated gelatin-degrading activity. (**C**) In vitro collagen degradation assay of y*Ci*CatB analyzed by SDS-PAGE. Lane M: protein marker; Lane 1: collagen only (2 h incubation, negative control); Lane 2: collagen + heat-inactivated y*Ci*CatB (2 h); Lane 3: collagen + E-64 pre-treated *Ci*CatB (2 h); Lane 4: collagen + active *Ci*CatB, 1 h incubation; Lane 5: collagen + active *Ci*CatB, 2 h incubation.

In the SDS-PAGE analysis, purified y*Ci*CatB was identified as a clearly visible individual band, and its molecular weight (50.3 kDa) was consistent with its predicted Size (Figure 1A). The serum anti-y*Ci*CatB IgG titre was 1:10^6^ following the final immunization, demonstrating that y*Ci*CatB induced immunogenicity. The results of the Western blot analysis showed that y*Ci*CatB was recognized by anti-y*Ci*CatB serum.

Gelatin zymography was performed to investigate the gelatin-hydrolyzing activity of y*Ci*CatB. A characteristic clear degradation band at approximately 50 kDa was detected by gelatin zymography (Figure 1B). These results indicate that y*Ci*CatB possesses gelatin-hydrolyzing enzymatic activity.

### 3.3. Localization of Natural CiCatB in Theronts

**Figure 2:**
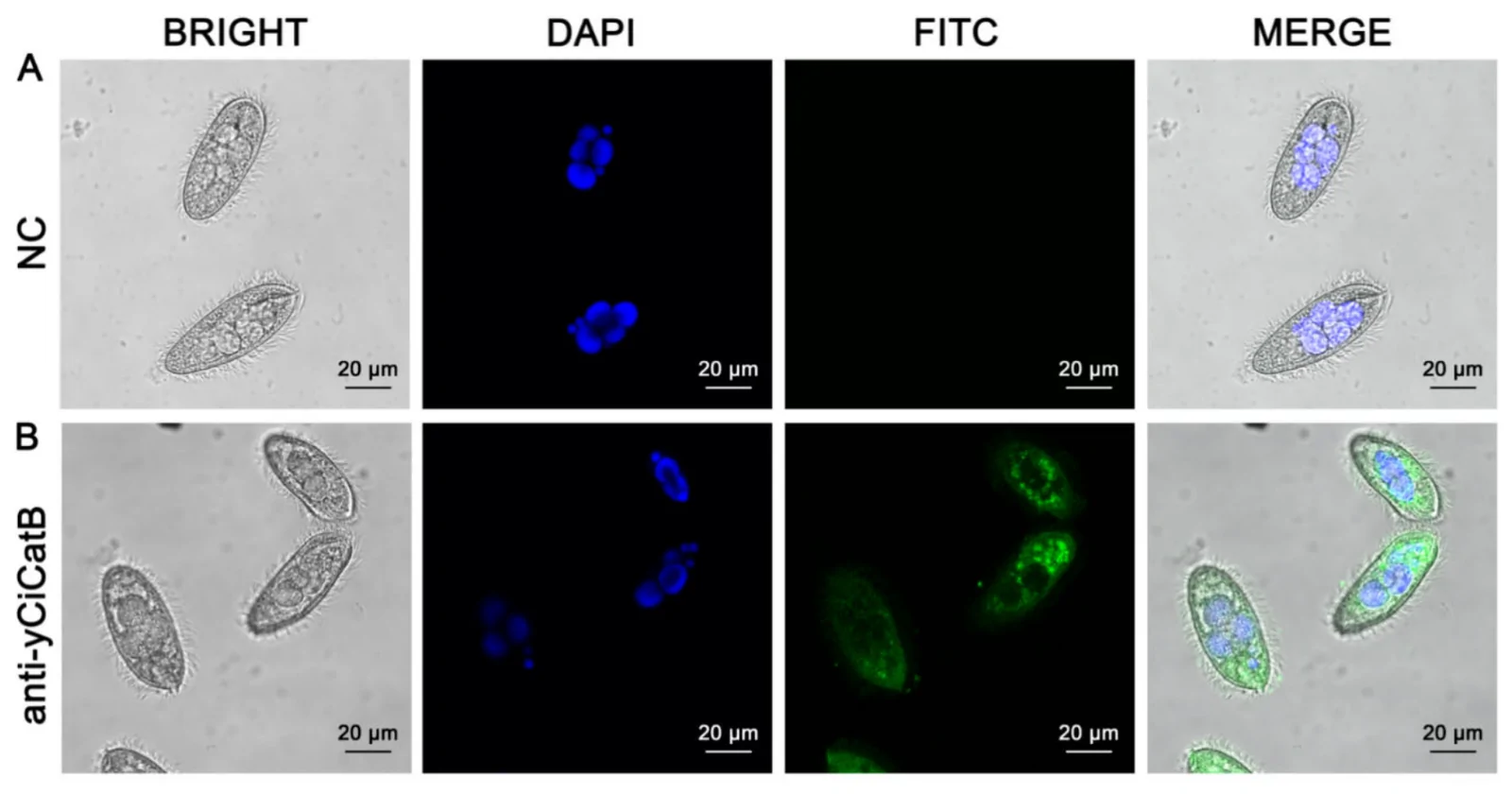
Localization analysis of *Ci*CatB in theronts by laser scanning confocal immunofluorescence. (**A**) Negative control group (NC): non-immunized mouse serum was used as the primary antibody; (**B**) anti-y*Ci*CatB serum was used as the primary antibody. Both groups were incubated with FITC-conjugated secondary antibody, and nuclei were counter-stained with DAPI. Green FITC fluorescence indicates y*Ci*CatB protein signals, and nuclei are visualized in blue (DAPI). Scale bars = 50 μm.

An IIF analysis was performed to assess the expression and localization of *Ci*CatB in theronts of *C. irritans*. Confocal microscopy showed *Ci*CatB mainly resided in the cytoplasm of theronts (Figure 2). Minor fluorescence staining was detected on the membrane edge and outside cells, which is consistent with bioinformatic predictions that *Ci*CatB may possess secretory properties. Nevertheless, these extracellular signals alone cannot confirm actual protein secretion, as they could also arise from cell damage or non-specific staining during sample preparation.

### 3.4. Enzyme Activity of yCiCatB

**Figure 3:**
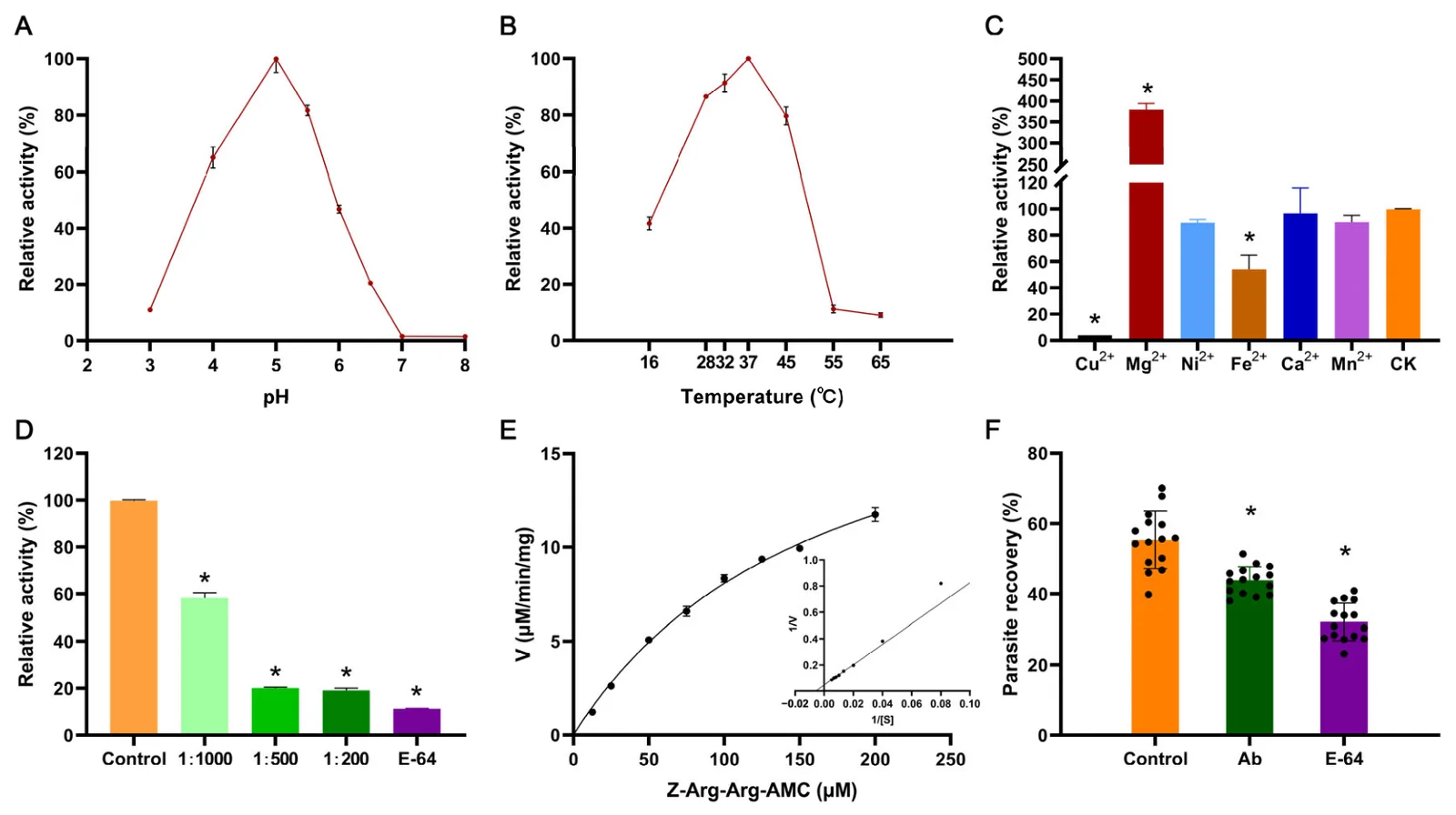
Biochemical characterization of y*Ci*CatB protease. (**A**) Effect of pH on the enzymatic activity of y*Ci*CatB. The relative activity was determined at pH 3.0–8.0. (**B**) Effect of temperature on y*Ci*CatB enzymatic activity. Relative activity was measured at temperatures anging from 16 °C to 65 °C. (**C**) Influences of various divalent metal ions (Cu^2+^, Mg^2+^, Ni^2+^, Fe^2+^, Ca^2+^, Mn^2+^) on y*Ci*CatB enzymatic activity. CK is the control without extra metal ions. (**D**) Inhibitory effects of serially diluted anti-*Ci*CatB immune serum (1:1000, 1:500, 1:200) and 10 μM E-64 on y*Ci*CatB activity. Control represents the reaction without serum or inhibitor. (**E**) Enzyme kinetic analysis of y*Ci*CatB toward substrate Z-Arg-Arg-AMC. The inset shows the Lineweave-Burk double-reciprocal plot. (**F**) Parasite recovery assay. Parasite recovery rates were compared among Control, antibody-treated (Ab), and E-64-treated groups. Each dot represents data from an individual fish. Kruskal–Wallis test was used for statistical comparison among groups. All data are presented as mean ± SD. Asterisks (*) indicate statistically significant differences compared with the corresponding control group (* *p* < 0.05).

The enzymatic activity of recombinant y*Ci*CatB was assayed using the fluorogenic substrate Z-Arg-Arg-AMC. We characterized the optimal pH and temperature for y*Ci*CatB catalytic activity. Maximal enzyme activity was observed at pH 5.0–5.5 (Figure 3A) and 37 °C (Figure 3B). These results indicated that y*Ci*CatB preferentially functions under acidic conditions. The effects of different divalent metal ions on the enzymatic activity of y*C*iCatB were evaluated. Compared with the control group (CK), Mg^2+^ significantly enhanced *yCi*CatB activity, raising the relative activity up to approximately 380%. In contrast, Cu^2+^ and Fe^2+^ exerted significant inhibitory effects. Cu^2+^ almost abolished the enzyme activity, while Fe^2+^ reduced relative activity to around 53%. Ni^2+^, Ca^2+^ and Mn^2+^ showed no significant influence on y*Ci*CatB catalytic activity (Figure 3C). Both E-64 inhibitor and anti-yCiCatB serum markedly reduced y*Ci*CatB enzymatic activity (*p* < 0.05), and the inhibitory effect of the antiserum occurred in a concentration-dependent manner (Figure 3D). The enzyme kinetic parameters of y*Ci*CatB were further determined by fitting substrate hydrolysis data to the Michaelis–Menten model (Figure 3E), yielding a Vmax of 21.63 μM/min/mg and a Km of 168.4 μM.

### 3.5. Degradation of Host Type I Collagen Protein yCiCatB

An in vitro substrate degradation assay was performed to determine whether activated y*Ci*CatB could hydrolyze host-derived type I collagen. SDS-PAGE analysis showed that the protein bands of host type I collagen almost disappeared after 1 h co-incubation with active y*Ci*CatB under the experimental conditions of pH 5.5 and 37 °C (Figure 1C). In the collagen-only control group, the electrophoretic profile of type I collagen remained intact throughout the incubation period. No obvious degradation fragments were detected in the group treated with heat-inactivated y*Ci*CatB, demonstrating that collagen hydrolysis strictly relied on the biological activity of y*Ci*CatB. When y*Ci*CatB was pre-incubated with the cysteine-protease-specific inhibitor E-64, its capacity to degrade type I collagen was completely abolished.

### 3.6. Effects of Protease Inhibitor E-64 and Anti-CiCatB Serum on Parasite Recovery

Parasite recovery was significantly reduced in both anti-*Ci*CatB serum (Ab) group and E-64 inhibitor groups compared with the untreated control group (*p* < 0.05) (Figure 3F). The mean parasite recovery in the control group reached 55.36%. The Ab treatment decreased parasite recovery to approximately 43.86%, while E-64 treatment produced a more pronounced reduction, lowering parasite recovery to around 32.12%. Note that non-immune mouse serum, which was used as negative control for immunofluorescence, was not included in this infectivity experiment, which is a limitation of the current assay. Thus, non-specific serum-derived effects cannot be completely excluded.

## 4. Discussion

Cathepsin B (CatB), a pivotal member of the cysteine protease superfamily, has been widely recognized as a key virulence factor in numerous protozoan and helminth parasites [36,37]. The CatB of the parasites such as *Toxoplasma gondii* participates in multiple pathogenic processes, including host tissue degradation, immune evasion, parasite adhesion and invasion, and intracellular survival, making it a research hotspot in parasitic pathogenic mechanisms and targeted drug development [38,39,40,41]. However, in contrast to terrestrial parasites, the molecular characteristics and functional mechanisms of CatB in *C. irritans* remain largely unexplored, with only very limited information available to date. In the present study, we successfully realized high-efficiency eukaryotic expression of codon-optimized *Ci*CatB via the *P. pastoris* system. Purified biologically active recombinant y*Ci*CatB protein and high-titer mouse anti-*Ci*CatB serum were obtained, which provides critical biological materials for subsequent functional verification and mechanism exploration.

Subcellular localization is closely correlated with the functional mode of parasitic proteases [42,43]. Existing studies have demonstrated that most functional CatBs of parasites are secretory proteins: they are synthesized in the cytoplasm, transported to the cell membrane, and finally secreted extracellularly to exert catalytic and pathogenic functions [44,45]. For instance, CatB from *T. spiralis* and *T. gondii* can be secreted outside cells to degrade host mucosal proteins and mediate parasitic invasion [27,29,40]. Similar to observations reported for other parasite-derived CatBs, laser confocal microscopy in the present study revealed that *Ci*CatB was mainly localized in the cytoplasm of *C. irritans* theronts, with partial signals detected at the cell-membrane periphery and in the extracellular space. This localization pattern is broadly consistent with bioinformatic predictions of potential secretory properties for *Ci*CatB, as well as reported functional features of protozoan parasite CatBs. Nevertheless, faint extracellular fluorescent signals from fixed samples cannot serve as definitive evidence for active protein secretion, since such signals may also originate from cell damage, protein leakage, or non-specific staining during sample preparation. Direct biochemical detection of *Ci*CatB in theront excretory-secretory products will be required to confirm whether *Ci*CatB functions as a secreted virulence-related effector.

Enzymatic activity and substrate specificity are the core functional basis of parasite CatB exerting pathogenic effects. Recent studies have reported that certain parasite CatBs exhibit potent hydrolytic activity against host structural proteins (e.g., keratin, elastin and collagen) [26,27], which is widely considered a crucial molecular mechanism enabling these parasites to breach host skin and mucosal barriers. In this study, the purified recombinant y*Ci*CatB exhibited significant protease activity, which could efficiently hydrolyze gelatin and type I collagen derived from large yellow croaker skin. This result is consistent with the functional characteristics of CatB from other parasites and further confirms the conserved pathogenic function of CatB in parasitic protozoa.

Parasite proteases possess enzymatic traits well-adapted to parasitic microenvironments and host infection contexts, which constitute a critical marker of parasite environmental fitness [46,47]. With the fluorogenic substrate Z-Arg-Arg-AMC, we comprehensively characterized the enzymatic properties of y*Ci*CatB, including pH and temperature preference, ion-dependent activity, kinetic parameters and inhibitory profiles. The enzyme exhibited the highest catalytic activity under weakly acidic conditions (pH 5.0–5.5), while neutral and alkaline environments significantly inhibited its enzymatic activity. This acidic pH aligns closely with the optimal conditions reported for other parasitic cathepsin B proteases [32,46]. For instance, recombinant cathepsin B from *Clonorchis sinensis* (*yCs*CBs) showed active enzymatic activity at pH 5.0–5.5 [32]. Considering the optimal acidic pH of recombinant *Ci*CatB in vitro, we hypothesize that an acidic microenvironment at the parasite–host contact site may favour *Ci*CatB enzymatic activity and thereby contribute to the invasion process [48]. Nevertheless, the local pH at this interface has not been directly measured in the current study, and this proposed mechanism remains to be experimentally validated. In terms of temperature adaptability, y*Ci*CatB showed peak activity at 37 °C and maintained high relative activity across the typical mariculture temperature range of 28–32 °C. This property might be partially related to the seasonal outbreak pattern of cryptocaryoniasis, but this hypothesis remains to be verified under field conditions [49,50]. In the present in vitro assay, Mg^2+^ markedly increased the catalytic activity of y*Ci*CatB, whereas Cu^2+^ nearly abolished enzyme activity, and Fe^2+^ also exerted significant inhibitory effects. These results characterize the metal-ion responses of purified recombinant *Ci*CatB under in vitro experimental conditions. Although copper preparations have been evaluated for the prevention and control of cryptocaryoniasis in several previous studies [51,52], it remains unclear whether Mg^2+^ functions as a physiological cofactor for *Ci*CatB in vivo, and whether environmental metal ions modulate the infectivity of C. irritans via *Ci*CatB activity. Collectively, our data provide supplementary biochemical insights into metal-ion responsiveness of parasite-derived cathepsin B. The kinetic parameters of y*Ci*CatB were determined as Km = 168.4 μM and Vmax = 21.63 μM/min/mg for the substrate Z-Arg-Arg-AMC, indicating only moderate affinity toward this short fluorogenic peptide. Nevertheless, our functional assays demonstrated that y*Ci*CatB exhibits potent hydrolytic efficiency toward native host Type I collagen from large yellow croaker skin. This apparent discrepancy is precedent among other parasitic cysteine proteases [46]. Short synthetic peptides are designed for in vitro enzyme quantification. They cannot simulate the complex spatial conformation and specific recognition motifs of physiological macromolecular substrates.

Both the general cysteine protease inhibitor E-64 and anti-y*Ci*CatB serum significantly reduced y*Ci*CatB enzymatic activity. E-64 is a well-established irreversible inhibitor of clan CA cysteine proteases and has been widely used to inhibit cathepsin B-like proteases from various parasites [26,53]. The potent inhibition by E-64 confirms that y*Ci*CatB belongs to the cysteine protease family and shares a conserved catalytic mechanism with other members of this class [36]. Functional blocking tests further revealed that parasite recovery was significantly reduced after inhibitor or antiserum intervention, which may imply a role of *Ci*CatB during host invasion by *C. irritans.* However, we cannot distinguish whether this outcome arises from blocked invasion or from reduced theront viability and motility after treatment. Thus, these results should be interpreted with caution. E-64 is a broad-spectrum cysteine protease inhibitor lacking strict specificity for *Ci*CatB, and non-immune mouse serum was not included as a control in the antiserum-based infectivity assay; thus non-specific effects cannot be fully excluded. From an application perspective, targeted suppression of CatB-like catalytic activity may interfere with parasite infection and colonization in fish hosts. Our in vitro observations indicate that *Ci*CatB represents a potential therapeutic target for developing inhibitory drugs against cryptocaryoniasis.

Nevertheless, the present findings are mainly obtained from in vitro biochemical and antibody-mediated neutralization assays, which provide only indirect evidence supporting the contribution of *Ci*CatB in pathogenicity. Future studies employing gene-editing approaches such as CRISPR/Cas9-mediated knockout or knockdown are warranted to generate *Ci*CatB-deficient parasite lines and directly verify its biological function [54,55]. Phenotypic characterization of these mutants, including evaluations of host-tissue invasion and in vivo infectivity, would allow rigorous assessment of the contribution of *Ci*CatB to parasite virulence. Additionally, the immunoprotective efficacy of *Ci*CatB as a vaccine antigen warrants further evaluation in fish models [18], including assessment of specific antibody titers, survival rates upon challenge infection, and histopathological changes, to determine its feasibility for subunit vaccine development.

## 5. Conclusions

In summary, we successfully expressed and purified recombinant *Ci*CatB via yeast expression system and systematically characterized its biochemical properties, including optimal pH/temperature conditions, ion responsiveness, and kinetic characteristics. Functional assays verified that y*Ci*CatB possesses prominent collagenolytic activity toward fish skin structural proteins. Functional blocking assays, using either cysteine protease inhibitor E-64 or anti-*Ci*CatB serum, significantly reduced parasite recovery. Collectively, this study suggests that *Ci*CatB may be involved in the host invasion process and contribute to the pathogenicity of cryptocaryoniasis. These findings offer preliminary insights for exploring CatB-targeted strategies to control this parasitic disease in marine aquaculture.

## 6. Patents

A recombinant protein of *Cryptocaryon irritans* cathepsin B (CiCatB) and its application. Applicant: Biotechnology Institute, Fujian Academy of Agricultural Sciences. Application No.: CN202611662399.9. Filing date: 26 October 2026. Inventors: H.C., Y.P., X.L., H.G. (pending patent).

## Data Availability

The data that support the findings of this study are available from the corresponding author upon reasonable request.

## Supplementary Materials

The following supporting information can be downloaded at: https://www.mdpi.com/article/10.3390/ani16213428/s1, Figure S1: Amino-acid sequence of signal-peptide-truncated *Ci*CatB and its codon-optimized variant.

## Abbreviations

The following abbreviations are used in this manuscript:

ELISA	Enzyme-linked immunosorbent assay
Z-Arg-Arg-AMC	Benzyloxycarbonyl-Arg-Arg-7-amino-4-methylcoumarin
DTT	Dithiothreitol
E-64	1-Trans-epoxysuccinyl-L-leucylamido-(4-guanidino) butane
PBS	Phosphate-buffered saline
DAPI	4′,6-Diamidino-2′-phenylindole
SDS-PAGE	Sodium dodecyl sulfate-polyacrylamide gel electrophoresis
EDTA	Ethylenediaminetetraacetic acid
