# Supplementary material for: Identification and Characterization of a Cathepsin B Protease in the Marine Fish Parasite Cryptocaryon irritans
Source: Animals (Basel). 2026 Oct 30;16(21):3428. doi: 10.3390/ani16213428 (PMC13762186; doi:10.3390/ani16213428)
Supplement: Supplementary file 1 [file animals-16-03428-s001.zip › animals-4576542-supplementary.pdf]

Protein	K I N F E E T K E I F V G I L E G A L A	20
Optimized	aagatcaacttcgaagagacaaaagagatcttcgtcggtatcttggagggtgctttggct	60
Protein	E A K M D K I A S C L K D D I K E G K L	40
Optimized	gaagctaagatggacaagatcgcctcctgtttgaaggacgacatcaaagagggtaagctg	120
Protein	I E E A I K D F L K E D F T S V K E G L	60
Optimized	atcgaagaggctatcaaggacttcctgaaagaggacttcacctccgtcaaagaaggtctg	180
Protein	K K L G E A I E N L P K A L S D C K A S	80
Optimized	aagaagttgggagaagccatcgagaacttgccaaaggctttgtctgactgtaaggcttcc	240
Protein	I E D I K K F Q Q M I M A F K S P I S Y	100
Optimized	Atcgaggacatcaagaaattccagcagatgatcatggccttcaagtccccaatttcctac	300
Protein	A F H V A K D I I V N G V N I Y H D I K	120
Optimized	gctttccacgttgccaaggacatcatcgttaacggtgtcaacatctaccacgacattaag	360
Protein	N T I N A Y Q T K E W E T M G F N I G H	140
Optimized	aacaccatcaacgcctaccagaccaaagaatgggagactatgggtttcaacatcggtcac	420
Protein	A L A S I L I G S S E I L K K D D S I Y	160
Optimized	gctttggcctctattttgattggttcctccgagatcctgaagaaggatgactctatctac	480
Protein	S H F M R E S P E E F A S K H L G L K I	180
Optimized	tcccacttcatgagagagtccccagaagagtttgcttccaagcacttgggtttgaagatc	540
Protein	V D L N E K F E R V D H R N L L Q Y I Q	200
Optimized	gtcgacctgaacgagaagttcgagagagttgaccacagaaacctgctgcagtacattcag	600
Protein	I P K F F D S R S Q W P D C I H P I R Q	220
Optimized	atcccaaagttcttcgactctagatcccaatggccagactgcattcacccaattagacaa	660
Protein	Q G H C G S C W A F S G S E V L S D R Y	240
Optimized	caaggtcactgcggttcttgttgggctttctctggttctgaagtcctgtccgacagatac	720
Protein	C I A S K E K I N L I L S P Q Y M V S C	260
Optimized	tgtatcgcctccaaagagaagatcaacctgattctgtccccacagtacatggtgtcctgt	780
Protein	D Y V D H A C K G G R L D Y L W Q F L E	280
Optimized	gattacgttgaccacgcctgtaaaggtggtagattggactacttgtggcagttcttggag	840
Protein	K K G I P T E D C D P Y V S G D G Q S M	300
Optimized	aagaagggtatcccaactgaggactgtgacccatacgtttctggtgacggtcaatctatg	900
Protein	P S C P S T C S D G T Q L R T L K A R R	320
Optimized	ccatcctgtccatctacttgttccgacggtactcagttgagaactctgaaggctagaaga	960
Protein	G S T K V F S D I K S I Q I E I M Q N G	340
Optimized	ggttccaccaaggttttctccgacatcaagtccatccagatcgagatcatgcagaacggt	1020
Protein	P V Q T G M M V Y D D F P Q Y Q A G K I	360
Optimized	ccagttcagaccggtatgatggtttacgatgacttcccacaataccaggccggtaagatc	1080
Protein	Y V P N S S K M L G G H A V K I V G W G	380
Optimized	tacgttccaaactcctctaagatgcttggtggtcacgccgttaagatcgttggttggggt	1140
Protein	N E N G V D Y W I V A N S W G P D W G E	400
Optimized	aacgaaaacggtgtcgactactggatcgttgctaactcttggggtccagattggggtgaa	1200
Protein	E G F F R I K M G E C N I D E D A I A G	420
Optimized	gaaggtttcttcagaatcaagatgggtgagtgcaacatcgacgaggatgctattgctggt	1260
Protein	I P I I S N L R P K Q F I Q	435
Optimized	atccccatcatttccaacttgaggcctaagcagttcatccaacat	1305

Figure S1: Amino-acid sequence of signal-peptide-truncated CiCatB and its codon-optimized variant.
